# Contactless actuation of perfluorinated ionomer membranes in salt solution: an experimental investigation

**DOI:** 10.1038/s41598-019-48235-9

**Published:** 2019-08-19

**Authors:** Alain Boldini, Maxwell Rosen, Youngsu Cha, Maurizio Porfiri

**Affiliations:** 1 Department of Mechanical and Aerospace Engineering, Tandon School of Engineering, New York University, 6 MetroTech Center, Brooklyn, NY 11201 USA; 2 Center for Intelligent & Interactive Robotics, Korea Institute of Science and Technology, Seoul, 02792 Republic of Korea; 3 Department of Biomedical Engineering, Tandon School of Engineering, New York University, 6 MetroTech Center, Brooklyn, NY 11201 USA

**Keywords:** Electrical and electronic engineering, Mechanical engineering, Chemical engineering

## Abstract

A variety of modeling frameworks have been proposed for ionic polymer metal composites (IPMCs), but the physical underpinnings of their actuation remain elusive. A critical step toward the validation of existing theories and transition to engineering practice entails the design of new experimental paradigms that could support hypothesis-driven research. While several factors exacerbate the complexity of experimenting with IPMCs, the presence of the electrodes plays a major role by hindering the repeatability of the results and bringing a number of difficult-to-measure parameters into the picture. Here, we seek to address these experimental confounds by investigating contactless actuation of perfluorinated ionomer membranes in salt solution. In contrast to IPMCs that bend toward the anode in response to an applied voltage, ionomer membranes display a consistent deflection toward the cathode. Through hypothesis-driven experiments where the membrane width, solution concentration, and voltage applied across the electrodes are systematically varied, we elucidate electrochemistry and mechanics of contactless actuation. The applied voltage and solution concentration have a dominant role on the electrochemistry, while mechanics is mainly affected by the applied voltage and membrane width. Our results depict a complex scenario, which is expected to inform future theoretical inquiries about IPMC actuation.

## Introduction

Ionic polymer metal composites (IPMCs)^1,2^ are a class of electroactive materials that hold promise as actuators for biomedical engineering^3^ and soft robotics^4,5^. Their large compliance, low actuation voltage, ability to experience large deformations, and potential to operate underwater have fueled research endeavors for more than twenty five years^1,2^. Recent breakthroughs in freeform fabrication^4,6^ are further expanding the reach of these electroactive materials, opening unprecedented opportunities for engineering their physical and geometric properties toward desired performance.

The main element of an IPMC is the ionomeric membrane, which consists of a porous polymeric material saturated with a solution^7^. Specifically, perfluorinated membranes such as NafionⓇ and AquivionⓇ are commonly used in IPMC manufacturing^2^. In these selectively-permeable membranes, anions are typically fixed, while cations can move through the pores^7^. In their original form discovered by Oguro and his team^8^, IPMCs comprise an ionomer core, plated by two noble metal electrodes and immersed in a solution. The application of a small voltage across the electrodes elicits a series of complex chemoelectromechanical phenomena, leading to the macroscopic bending of the actuator^1,2^.

Several theories have been proposed to explain IPMC actuation over the past twenty years. The seminal studies of Nemat-Nasser and Li^9^ and de Gennes *et al*.^10^ identified charge redistribution as the main driver of IPMC actuation, within the different domains of micromechanics and linear irreversible thermodynamics, respectively. It is generally recognized that this phenomenon, experimentally confirmed through fluorescence microscopy^11^, elicits a differential osmotic pressure near the two electrodes that causes the macroscopic bending toward the anode. Building upon these efforts, several other modeling frameworks have been put forward, spanning phenomenological and physically-based perspectives. Some authors have proposed black box models for IPMC actuation^12–14^, in which the transduction principle of IPMCs is modeled through a two-port element transforming electrical into mechanical variables, and vice versa. Other authors have focused on physically-based theories of IPMCs, encompassing models grounded in micromechanics^15^, theory of mixtures^16^, Poisson-Nernst-Planck systems^17–19^, and theory of porous media^20^.

Recently, our group has proposed a thermodynamically-consistent continuum model to describe mechanics and electrochemistry of IPMCs^21^. The main element of novelty of the model is the presence of Maxwell stress tensor^22^, whose interaction with osmotic pressure is hypothesized to determine IPMC actuation. Particularly enticing to this interaction is the possibility to explain the phenomenon of back-relaxation from first physical principles. Experimentally observed for the first time by Asaka and colleagues^23^, this phenomenon consists of fast bending toward the anode, followed by slow relaxation toward the cathode, upon the application of a step voltage. This surprising effect is commonly associated with the so-called added mass, whereby solvent molecules in the ionomer are first dragged toward the anode by counterions migration and then slowly diffuse back to drive the relaxation of the IPMC^24^. This explanation presents some inconsistencies when compared with experimental observations^16,25,26^, which could be partially resolved by embracing our thermodynamically-consistent continuum model as we have demonstrated in previous efforts^27,28^. While we are able to resolve some of the qualitative discrepancies of the added mass explanation with respect to existing experiments, it is difficult to confidently validate any theory of IPMC actuation and propose which approach should be preferred when designing IPMC actuators.

Two main factors challenge the validation and limit the predictive power of existing theories. First, these theories are based on a large number of parameters that cannot be individually identified^2,24^, thereby masking their individual role on IPMC actuation. Second, these theories are based on a simplified description of the electrodes, where charge accumulation should take place, such that small variations in geometric and physical properties could translate into macroscopic variations in IPMC actuation, as we have shown, for example, in the study of IPMC impedance^29,30^. A potential avenue to mitigate these drawbacks is offered by the experimental paradigm established by Kim and colleagues^31^, where a plain ionomer sample is placed in a salt solution between two external electrodes.

**Figure 1:**
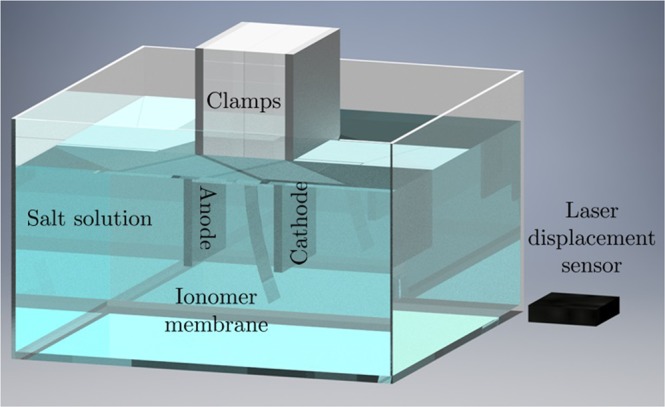
Sketch of the setup used for our experiments, analogous to the setup by Kim and colleagues^31^. A cantilevered ionomer membrane is positioned between two graphite electrodes. The assembly is immersed in a salt solution. A voltage is applied across the electrodes to create an electric field that elicits the deformation of the membrane. A laser displacement sensor is used to measure the tip displacement of the membrane.

Experiments were carried on one cantilevered sample for a fixed actuation voltage and a single solution. By recording the time trace of the tip displacement at varying actuation frequencies, the authors discovered a surprising deflection of the sample toward the cathode, rather than the anode, as one would expect from traditional IPMCs. In other words, without the electrodes, the response of the actuator seemed to mirror an IPMC experiencing back-relaxation. The main objective of this paper is to systematically examine this claim in a statistically-based experimental design, where we test multiple geometries, solutions, and actuation voltages. In addition to the tip displacement, we synchronously acquire the time trace of the current through the external electrodes to help understand the electrochemical drivers of the actuation. The setup of our experiment is analogous to the one of Kim and colleagues^31^, as shown in Fig. 1.

This setup shares similarities with classical work in the literature of polyelectrolyte gels, where contactless actuation with external electrodes has been widely investigated, even before the advent of IPMCs. For instance, Shiga and Kurauchi^32^ studied the deformation of polyacrylamide (PAA) gels in distilled water under a constant voltage applied across external electrodes. The response of these hydrogels varied with the concentration of polyions in the gel. Specifically, PAA gels deformed toward the cathode and slowly relaxed toward the anode for low polyion concentrations, while they deflected without relaxation for higher concentrations. When polyions were not present in the hydrogel, no deflection was observed. Similarly, Grimshaw and colleagues^33^ demonstrated swelling of polymethacrylic acid (PMAA) under the effect of an external electric field, which can be exploited to perform mechanical work.

However, these experimental studies present relevant differences with respect to contactless actuation of perfluorinated ionomer membranes. First of all, perfluorinated membranes^34^ and hydrogels^35^ have different structures, which strongly affect their water uptake. While hydrogels can reach hydration levels of more than 100% in terms of weight of water per weight of dry polymer^36^, perfluorinated membranes present cluster morphologies that limit their water uptake (less than 45% in Nafion in standard conditions^37^). In addition, previous efforts on hydrogels focused on relatively high voltages^32^ or high current densities^33^, which could lead to highly nonlinear electrochemistry, likely more complex than that underlying the actuation of perfluorinated ionomer membranes in the setup of Kim and colleagues^31^. Finally, the actuation time in hydrogel experiments was on the order of minutes^32^, while the mechanical response of perfluorinated membranes was on the order of seconds^31^.

In the absence of the ionomer sample, the experimental setup corresponds to a classical electrochemical cell^38^, whose working principle underpins, among the others, electrolytic cells^39^, fuel cells^40^, batteries^41^, and electrochemical capacitors^42^. In an electrochemical cell, salt is dissolved in water to form an electrolyte solution^38^, which can take several forms – here, we consider a binary electrolyte where the species have the same valency and a comparable diffusivity. From the seminal work of Helmholtz^43,44^ and Gouy^45^, it is known that charge boundary layers develop in the vicinity of the electrodes, forming two thin double layers with a thickness of the order of the Debye screening length *l*^46^. The latter is a function of the dielectric constant *ε* of the solution, the temperature ${\mathscr{T}}$, and the electrolyte concentration *C*_0_, according to $\lambda =\frac{1}{ {\mathcal F} }\sqrt{\frac{\varepsilon {\mathscr{R}}{\mathscr{T}}}{2{C}_{0}}}$, where ${\mathcal F}$ and ${\mathcal R}$ are the Faraday constant and the universal gas constant, respectively^46^. The characteristic time for the charges to migrate toward the electrodes is $\tau =\frac{\lambda h}{{\mathscr{D}}}$, where ${\mathscr{D}}$ is the diffusivity of the ions in the solution, and *h* is the semigap between the electrodes^46^.

For low voltages, of the order of the thermal voltage (25.9 mV at room temperature), charge dynamics in the solution bulk and pile-up at the electrodes is captured by a simple *RC* model^46^, where the effective conductivity and capacitance per unit surface area are, respectively,

1a $$\sigma =\frac{\varepsilon }{2\lambda \tau }=\frac{{\mathscr{D}}{ {\mathcal F} }^{2}{C}_{0}}{h {\mathcal R} {\mathscr{T}}},$$

1b $$\gamma =\frac{\varepsilon }{2\lambda }= {\mathcal F} \sqrt{\frac{\varepsilon {C}_{0}}{2 {\mathcal R} {\mathscr{T}}}}\mathrm{.}$$

Although theoretical insight is limited to moderately low voltage levels, it is tenable to assume that increasing the voltage has a secondary effect on the effective resistance, while it leads to a nonlinear increase in the capacitance^46^. The concentration of the solution offers an extra degree of freedom to the problem by allowing for changing the time scale of the pile-up and the migration, without altering the underpinning phenomenon.

Placing an ionomer sample in the electrochemical cell, as in Fig. 1, should hinder charge dynamics in the bulk of the solution and promote the formation of additional electric double layers at the ionomer-solution interface due to the difference in their electrochemical properties, the so-called Donnan potentials^47^. The first phenomenon should produce an increase in the effective resistance, as a function of the width of the ionomer sample. The second phenomenon should manifest into a reduction of the effective capacitance of the electrochemical cell, since the additional double layers would cause a secondary capacitance, in series with the original double layer capacitance. Based on these arguments and the dependence of *σ* and *γ* on the physical parameters of the solution for moderate voltages in Equation (1), we formulated the following hypotheses: (H1) irrespective of the presence/width of the sample and the solution concentration, a higher voltage would produce both higher peak current and charge stored at the electrodes; (H2) irrespective of the solution concentration and the applied voltage, the presence of the ionomer would reduce both the peak current and the charge stored at the electrodes, and the extent of this reduction would be higher for wide samples; and (H3) irrespective of the presence/width of the sample and the applied voltage, a higher solution concentration would produce both higher peak current and charge stored at the electrodes.

The motion experienced by the ionomer is mediated by the fluid-structure interaction with the surrounding solution^48–51^. For small oscillations of the ionomer, the fluid-structure interaction is controlled by the geometry of the sample, such that increasing the width will produce an increase in the added mass effect. As the sample oscillates in the solution, it will displace a portion of the surrounding solution, roughly corresponding to a cylinder of diameter equal to the width of the sample, such that the added mass is

2 $${m}_{{\rm{add}}}={\rho }_{f}\frac{\pi {b}^{2}L}{4},$$

where *ρ*_*f*_ is the mass density of the solution, and *b* and *L* are the width and length of the sample, respectively. This added mass effect should control the time scale of the actuation, rather than the extent of the deflection, which will be regulated by the concurrent electrochemical response. The higher is the applied voltage, the higher should be the internal actuation, such that the sample will experience a larger deflection^1,2^. Based on these arguments, we formulate the following two additional hypotheses: (H4) irrespective of the solution concentration and the applied voltage, the time required to reach the peak displacement would be higher for wide samples; and (H5) irrespective of the width of the sample and the solution concentration, a higher voltage would produce higher peak values of the tip displacement.

The features extracted from the measurements collected during our experiments were statistically analyzed to elucidate which variables have a significant effect on the electrochemistry of the overall system and on the actuation of the ionomer membranes. These factors were then compared with the proposed hypotheses to assess their validity, thereby seeking to clarify our findings in a physical framework.

## Results

Overall, we analyzed twelve samples cut from the same Nafion N117 membrane, six with a width of 5 mm (“thin” membranes) and six with a width of 40 mm (“wide” membranes). Each sample was tested in two solutions of sodium chloride, with concentrations of 0.1 M and 0.5 M. For each width, we characterized three membranes at the lower (higher) concentration first and at the higher (lower) concentration later. During an experimental trial, twenty step inputs of one minute in duration each were applied at the electrodes, separated by equal intervals of one minute when the electrodes were shorted. The polarity of the voltage during a trial was alternated between the one-minute pulses to mitigate potential asymmetries associated with prebending of the sample. The voltage of each of the step inputs was randomized between 0.5 V and 1 V, for a total of twenty step inputs where the voltage value and the polarity were fully balanced. The applied voltage was considerably less than the voltage in the experiments performed by Kim and colleagues^31^ to avoid water electrolysis and bubbles’ formation.

Accounting for any possible combination of membrane width (thin or wide), solution concentration (0.1 M or 0.5 M), and applied voltage (0.5 V or 1 V), we ultimately considered eight experimental conditions. To control for the presence of the membranes, an equal number of trials with an equivalent randomization procedure was performed without the membranes, thereby adding four more control conditions to the experimental design for the four possible combinations of solution concentration and applied voltage. The twelve conditions performed in this work are listed in Table 1. In addition, we performed some control tests with the ionomer membranes in deionized (DI) water or with ion-blocking mylar membranes in salt solution, see Supplementary Information.

**Table 1:** Mean values and standard errors (in parentheses) of the response variables for each experimental condition.

Conditions			Features extracted			
Width	Molarity [M]	Voltage [V]	Peak current [A]	Charge [C]	Time to reach peak displacement [s]	Peak displacement [*μ* m]
Solution	0.1	0.5	0.0751 (0.0008)	0.0061 (0.0000)	NA	NA
Solution	0.1	1	0.1644 (0.0017)	0.0155 (0.0002)	NA	NA
Solution	0.5	0.5	0.0996 (0.0017)	0.0067 (0.0001)	NA	NA
Solution	0.5	1	0.2294 (0.0034)	0.0162 (0.0002)	NA	NA
Thin	0.1	0.5	0.0753 (0.0007)	0.0062 (0.0001)	0.6479 (0.0177)	29.2382 (1.4374)
Thin	0.1	1	0.1644 (0.0016)	0.0155 (0.0002)	0.6773 (0.0194)	61.3270 (2.3871)
Thin	0.5	0.5	0.0989 (0.0016)	0.0069 (0.0001)	0.5881 (0.0177)	22.7745 (1.2696)
Thin	0.5	1	0.2295 (0.0034)	0.0162 (0.0002)	0.5898 (0.0084)	59.9663 (2.3843)
Wide	0.1	0.5	0.0750 (0.0007)	0.0062 (0.0001)	0.7783 (0.0300)	22.7464 (1.6733)
Wide	0.1	1	0.1644 (0.0016)	0.0156 (0.0002)	0.8076 (0.0301)	51.9413 (3.0034)
Wide	0.5	0.5	0.0974 (0.0015)	0.0068 (0.0001)	0.6871 (0.0212)	24.5806 (1.8750)
Wide	0.5	1	0.2251 (0.0031)	0.0160 (0.0002)	0.7120 (0.0211)	58.2381 (2.9976)

NA indicates not available data, corresponding to the four control conditions in the absence of the ionomer membrane.

**Figure 2:**
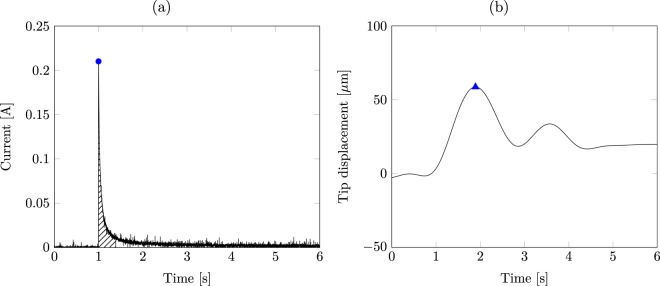
Representative response for a wide membrane, at the solution concentration of 0.5 M, tested at 1 V: (**a**) time trace of the current, and (**b**) time trace of the tip displacement. The step voltage is applied at time *t*_0_ = 1 s. In (**a**), the blue dot represents the peak current, while the hatching indicates the area integrated to obtain the total charge. In (**b**), the blue triangle indicates the peak displacement.

For all the tested samples, we confirmed the findings of Kim and colleagues^31^, whereby the samples always move toward the cathode as a result of the applied voltage. Figure 2 illustrates typical experimental time traces of the current through the external electrodes and tip displacement of the ionomer membrane. Similar to an IPMC^52^, the current monotonically decays from a peak value to almost zero as time progresses. The tip displacement increases rapidly to reach its peak value and then slowly decays to zero while the voltage is still applied, albeit non-monotonically. The decay of the tip displacement is characterized by a slower time scale compared to the current, suggesting that electrochemistry is faster than the mechanical response. In the figure, we mark the features that were extracted from the data as response variables for the statistical analysis: the peak current, total charge, peak displacement, and time to reach the peak displacement. The peak current was computed as the maximum value of the current, following the application of the step input across the electrodes. The total charge was computed by integrating the time trace of the current from its peak to the time when it reached 5% of the peak. The peak displacement was obtained as the maximum value of the time trace of the tip deflection during the first five seconds of the response. The time to reach the peak displacement was calculated as the time lapse between the peak in the tip displacement and the peak current.

The data were fitted into a mixed-effects model, with width, concentration, and voltage as the explanatory variables, and peak current, total charge, time to reach the peak displacement, and peak displacement as the response variables. All the explanatory variables were treated as categorical, such that concentration and voltage had two levels, while the width had three levels to encompass the control conditions where the membrane was not present. To avoid pseudoreplications in the statistical analysis, the identity of each membrane was included in the model as a random effect. For the peak current, total charge, and time to reach the peak displacement, a generalized linear mixed-effects model (GLMM) with Gamma errors was fitted, to normalize the residuals, while a linear mixed-effects model (LMM) was fitted for the peak displacement, whose residuals were already normal. For each response variable, we started fitting from a full model, based on our hypotheses, with triadic interactions^53^. Backward elimination was applied to obtain the most parsimonious model, minimizing the second-order Akaike Information Criterion (AICc), a correction to the standard AIC for small sample size^54^. The fixed factors in the most parsimonious model for each response variable are listed in Table 2. We then performed three-way analysis of variance (ANOVA) tests to obtain the significant explanatory variables and interactions in the most parsimonious model^53^, whose results are shown in Table 2. For each explanatory variable, post-hoc analyses, through Tukey’s honest significant difference test (HSD)^53^, were conducted on the most parsimonious model to identify pairwise significant differences, by aggregating with respect to non-significant variables and interactions.

**Table 2:** Results of ANOVA test for each explanatory variable and interaction term in the most parsimonious model for all the response variables. *df* indicates the degrees of freedom of the *χ*^2^ distribution. Values in bold identify statistically significant results (p < 0.05).

Fixed factors	*χ* ^2^	*df*	*p*
**Peak current (GLMM)**			
Width	0.1058	2	0.949
Concentration	2074.64	1	<**0.001**
Voltage	13,566	1	<**0.001**
Concentration:Voltage	172.1	1	<**0.001**
**Charge (GLMM)**			
Width	0.049	2	0.976
Concentration	65.27	1	<**0.001**
Voltage	20,016	1	<**0.001**
Concentration:Voltage	84.57	1	<**0.001**
**Time to reach peak displacement (GLMM)**			
Width	12.69	1	<**0.001**
Width:Concentration	36.28	2	<**0.001**
Width:Voltage	5.124	2	0.077
**Peak displacement (LMM)**			
Voltage	468.5	1	<**0.001**
Voltage:Width	7.211	2	**0.027**
Voltage:Concentration	10.20	2	**0.006**
Voltage:Width:Concentration	30.24	2	<**0.001**

Mean values with standard errors for each of the response variables in all the conditions are shown in Table 1. Each quantity was computed over 60 independent measurements, corresponding to six membranes and ten one-minute pulses at a given value of the applied voltage and solution concentration.

**Figure 3:**
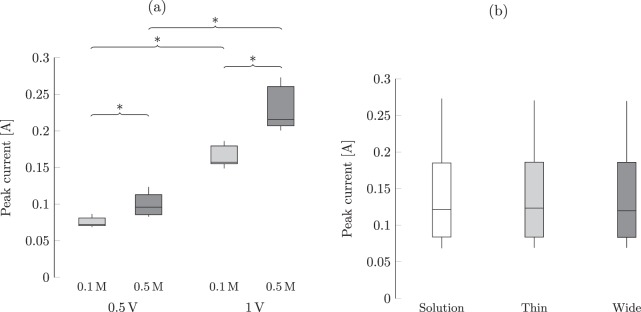
(**a**) Effect of the solution concentration and applied voltage on the peak current through the electrodes, and (**b**) effect of the width on the peak current. The band inside each box indicates the median, and the bottom and top of the box identify the first and third quartiles, respectively. The whiskers delimit the 1.5-interquartile range of the data, and the crosses are realizations out of this range. A significant difference (*p* < 0.05) from post-hoc comparisons of conditions with only one different explanatory variable is indicated through braces with an asterisk.

From the ANOVA test in Table 2, we found a significant interaction of the voltage with the concentration explaining the variation in peak current. Post-hoc pairwise comparisons revealed that the conditions at low voltage significantly differed from those at high voltage for each width and solution concentration tested (*p* < 0.001 for all), as shown in Fig. 3(a). In agreement with our predictions, we determined that an increase of the applied voltage consistently resulted into a significant increase in the peak current, see Fig. 3(a). In addition, from post-hoc comparisons, for each width and voltage tested, we recorded an increase in the peak current following an increase in the solution concentration (*p* < 0.001 for all). No significant effects of the presence and width of the sample on the peak current were recorded, as shown in Fig. 3(b).

**Figure 4:**
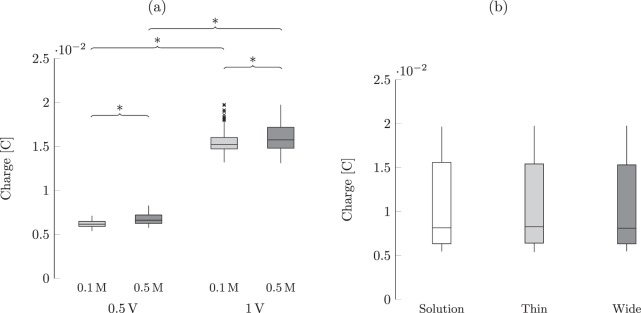
(**a**) Effect of the solution concentration and applied voltage on the total charge stored at the electrodes, and (**b**) effect of the width on the charge. The band inside each box indicates the median, and the bottom and top of the box identify the first and third quartiles, respectively. The whiskers delimit the 1.5-interquartile range of the data, and the crosses are realizations out of this range. A significant difference (*p* < 0.05) from post-hoc comparisons of conditions with only one different explanatory variable is indicated through braces with an asterisk.

The results for the total charge stored at the electrodes mirrored those for the peak current, whereby the solution concentration and applied voltage interacted to explain the variation in the total charge. Post-hoc comparisons of conditions with the same width and concentration indicated a strong dependence on the applied voltage (*p* < 0.001 for all), whereby increasing the voltage elicited a robust charge increase, as shown in Fig. 4(a). In accordance to our expectations, we determined that an increase in the concentration for the same width and voltage elicited a significant increase in the total charge (*p* < 0.001 for all in post-hoc analyses), see Fig. 4(a). Although such an increase reaches a level of statistical significance, we acknowledge that it consists of only a fraction of a millicoulomb. Similar to the analysis of the peak current, we did not identify an effect of the presence and width of the sample, as shown in Fig. 4(b).

**Figure 5:**
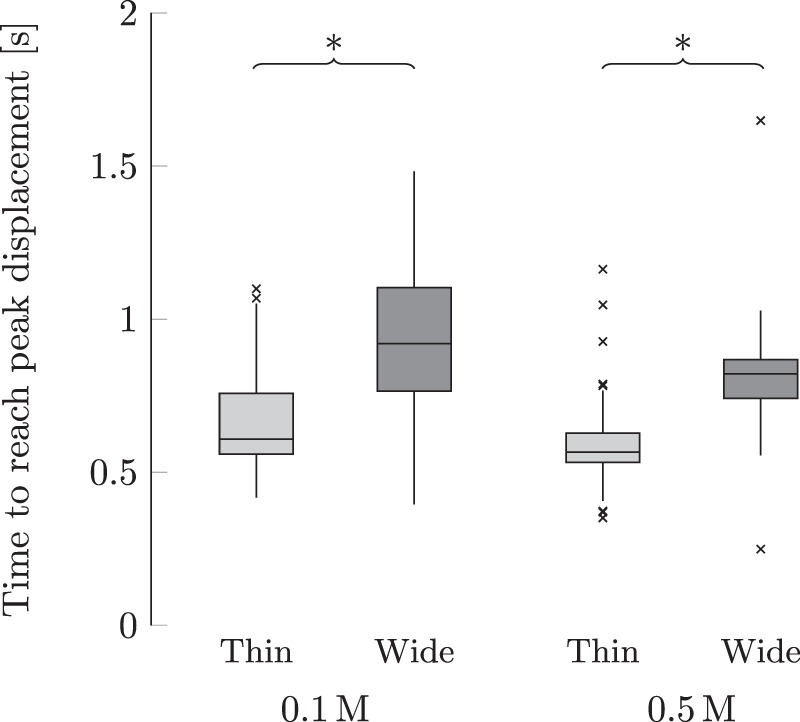
Effect of the membrane width and solution concentration on the time to reach the peak displacement. The band inside each box indicates the median, and the bottom and top of the box identify the first and third quartiles, respectively. The whiskers delimit the 1.5-interquartile range of the data, and the crosses are realizations out of this range. A significant difference (*p* < 0.05) from post-hoc comparisons of conditions with only one different explanatory variable is indicated through braces with an asterisk.

From ANOVA tests, we registered a significant dependence of the time to reach the peak displacement on the interaction between the width of the sample and the solution concentration. In accordance with our expectations, for given solution concentration and applied voltage, an increase in the width of the sample resulted into a robust increase in the time to reach the peak displacement (*p* < 0.004 for all in post-hoc analyses). While the extent of this change depended upon the solution concentration, see Fig. 5, we failed to identify a triadic interaction of the width with the solution concentration and applied voltage.

**Figure 6:**
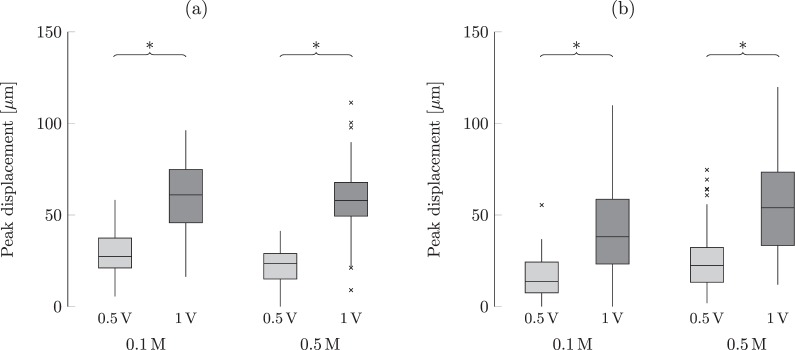
Effect of the solution concentration and applied voltage on the peak displacement of the membrane for a given width of the membrane: (**a**) thin, and (**b**) wide. The band inside each box indicates the median, and the bottom and top of the box identify the first and third quartiles, respectively. The whiskers delimit the 1.5-interquartile range of the data, and the crosses are realizations out of this range. A significant difference (*p* < 0.05) from post-hoc comparisons of conditions with only one different explanatory variable is indicated through braces with an asterisk.

We observed that the peak displacement was explained by the triadic interaction of the voltage with the sample width and solution concentration. As one should expect, for given width and concentration, post-hoc pairwise comparisons revealed a significant increase of the peak displacement with an increase in the voltage (*p* < 0.001 for all). The extent of this change is mediated by both the sample width and solution concentration, as shown in Fig. 6.

In addition to the experimental conditions listed in Table 1, we tested ionomer membranes in DI water and mylar membranes in salt solution, as summarized in the Supplementary Information.

## Discussion

In this paper, we examined contactless actuation of perfluorinated ionomer membranes in salt solution. We systematically explored different combinations of the width of the membrane, concentration of the solution, and applied voltage to elucidate the electrochemistry of the system and the actuation of the membranes. We formulated the following five hypotheses:

1. irrespective of the presence/width of the sample and the solution concentration, a higher voltage would produce both higher peak current and charge stored at the electrodes;
2. irrespective of the solution concentration and the applied voltage, the presence of the ionomer would reduce both the peak current and the charge stored at the electrodes, and the extent of this reduction would be higher for wide samples;
3. irrespective of the presence/width of the sample and the applied voltage, a higher solution concentration would produce both higher peak current and charge stored at the electrodes;
4. irrespective of the solution concentration and the applied voltage, the time required to reach the peak displacement would be higher for wide samples;
5. irrespective of the width of the sample and the solution concentration, a higher voltage would produce higher peak values of the tip displacement.

Our results support hypothesis H1, whereby we consistently registered an increase of the peak current and total charge with the voltage, while keeping the same width and solution concentration. These observations are in agreement with the predictions of the classical *RC* circuit used to model electrochemical cells, even if the voltage considered in our experiments is far larger than the range for which this approximation should hold quantitatively^46^. In particular, the increase of the peak current is explained by the fact that the effective resistance is, to a first degree of approximation^46^, independent of the applied voltage, as shown in Equation (1a), such that a higher step input voltage leads to a higher peak current. On the other hand, the total charge stored at the electrodes depends only on the capacitance, which increases nonlinearly with the applied voltage^46,55^. As first suggested by Chapman^55^, the approximation of constant capacitance for the electric double layers in Equation (1b) should be corrected for moderately high voltages^46^ by including the effect of the voltage drop *ζ* across the diffuse layer (the so-called “zeta potential”) through $\gamma =\frac{\varepsilon }{\lambda }\,\cosh (\frac{ {\mathcal F} \zeta }{ {\mathcal R} {\mathscr{T}}})$. We expect that a similarly nonlinear relationship holds in our experiment, even if the large value of the applied voltage strains the validity of this formula^46^ and steric effects could drastically affect charge dynamics and the development of the electric double layers^56,57^.

With respect to hypothesis H2, we failed to detect any effect of the presence and width of the membranes on either the peak current or the charge stored. While we cannot exclude that this may be simply related to limited statistical power of our experimental study, we should also contemplate physically-based explanations. A possible explanation could be that the effective electric resistance of the ionomer membrane is comparable to the resistance of the solution, such that the presence of the membrane would not constitute an obstacle to the movement of the ions. As the effective resistance in the electrolyte^46^ and in the ionomer^58^ have analogous expressions, this explanation could be tenable if the smaller diffusivity in the ionomer is compensated by a higher charge concentration^9^, see Equation (1a). An alternative explanation could be that the presence of the membrane favors the movement of ions along multiaxial branches in the solution, which outweighs the potential increase in the effective resistance associated with the movement through the membrane. This second explanation seems to be supported by the results of the tests on ion-blocking mylar membranes (see Supplementary Information), where we also failed to identify an appreciable reduction in the peak current as a function of the width of the membrane, at least for the higher concentration.

Hypothesis H3 is supported by the results of our experiments, whereby we registered an increase in the value of the peak current and the total charge in response to an increase in the concentration of the solution. This twofold effect can be explained through Equation (1). In fact, an increase in the concentration of the solution elicits a reduction of the bulk resistance, related to the higher availability of charge carriers^46^, as shown in Equation (1a), yielding an increase of the peak current. In addition, the capacitance of the electric double layers at the electrode surface increases with an increase in the concentration, due to the reduction of the Debye screening length^46^, see Equation (1b). This effect should produce the observed increase in the total charge.

The results of our experiments support the validity of hypothesis H4, whereby an increase in the width of the membrane elicited a considerable increase in the time to reach the peak displacement, for the same concentration and voltage. This quantity can be related to the inverse of the fundamental frequency of the sample^59^. If the experiments were performed in air, we would not register any effect of the width on the natural frequency, given that both the stiffness and mass have a linear dependence on the width. The presence of the surrounding solution induces a nonlinear dependence of the mass on the width^48–51^. The mass can be associated with a cylinder of water with diameter equal to the width of the membrane and height equal to the length of the membrane, as shown in Equation (2). As a result, we should expect that the time to reach the peak displacement scales with the inverse of the square root of the width of the sample. Due to the physical proximity of the membrane to the electrodes and three-dimensional effects, we should also expect that the fluid confinement will modulate the dependence of the modal mass on the width^60^, potentially altering Equation (2).

Our experiments are also in support of H5, as we registered an increase of the peak displacement due to an increase of the voltage, for the same width and concentration. The relationship between applied voltage and peak tip displacement seems to be linear, where a twofold increase in the voltage (0.5 V to 1 V) elicits a twofold increase in the peak displacement (25–30 *μ*m to 50–60 *μ*m). This result indicates that actuation is controlled by current and charge in the system, suggesting a direct coupling between electrochemical quantities and actuation. However, due to the lack of detailed electrochemical measures in the membrane, we cannot delve into physical explanations of this relationship. In fact, presently we have no access to either the time trace of the current through or the voltage across the ionomer membrane. Should the membrane permit ion motion like the solution, we would expect the electrochemical response of the ionomer to be similar to the response of the solution. In this case, the registered peak current and charge stored would capture the charge dynamics in the ionomer and explain its actuation. On the other hand, should the membrane block ion movement and elicit multiaxial branches of ion motion in the solution, it would be difficult to relate the electrochemical response of the membrane to the measured time traces for the solution. Future work should seek to delve into this aspect through computer simulations of the system electrochemistry in three-dimensions. Beyond challenges in the numerical implementation of the model, key challenges will be posed by the selection of proper boundary conditions at the ionomer-solution interface. Perhaps, experiments presented in this paper will help identify realistic boundary conditions to clarify actuation of ionomer membranes, thereby informing an improved understanding of IPMC actuation.

Overall, these results depict a complex picture that needs to be clarified to elucidate the physical underpinnings of the actuation of ionomer membranes in salt solution, expanding on the observations made by Kim and colleagues^31^. As advocated by the authors, a potential explanation for this phenomenon is differential electroosmotic drag^61,62^, whereby Nafion is a selectively-permeable membrane which allows the transport of cations, while blocking anions^1^. This explanation shares similarities with the theory proposed by Shiga and Kurauchi^32^ and Grimshaw and colleagues^63^, where the motion of counterions through the ionomer could elicit a gradient in the osmotic pressure that would cause bending of the membrane. However, our preliminary numerical simulations^64^ do not seem to support this explanation, whereby osmotic effects alone would only yield modest bending toward the anode. These simulations were based on a Poisson-Nernst-Planck model of the electrochemistry that excluded the motion of the solvent, the presence of Stern layers at interfaces, and water dissociation, to name just a few of the key simplifications^65^ of the approach.

An alternative explanation might entail early back-relaxation of the samples^27^, in the absence of metal electrodes that could block the movement of the counterions and solvent across the ionomer-solution interface. The preliminary simulation results in our previous work^64^ seem to support this explanation, whereby Maxwell stress generated by large electric fields at the ionomer-solution interface would lead to a consistent bending toward the cathode. However, the numerical values of the bending moment associated with Maxwell stress are unlikely to reproduce tip displacements of the order of one hundred microns. We expect that expanding the computational model to overcome some of the present simplifications could help reduce the discrepancy between numerical and experimental results.

While this experimental study cannot settle the explanation, due to the impossibility of measuring electrochemical variables in the membrane, it can serve as a validation benchmark for high-fidelity simulations of the whole chemoelectromechanical system, which could pinpoint the physical drivers of actuation. Additional testing with this experimental setup, considering different counterion forms in the ionomer and in the solution, may help elucidate the physical underpinnings of contactless actuation of perfluorinated membranes in salt solution, disentangling the critical factors underlying the electrochemistry and mechanics of the system.

## Methods

In this experiment, we investigated contactless actuation of perfluorinated ionomer membranes in salt solution, following the application of a voltage input across external electrodes. During experimental trials, we measured the tip displacement and current through the electrodes to elucidate the concurrent electrochemistry and mechanics of actuation. From our measurements, we extracted several features used as response variables in the statistical analysis.

### Ionomer samples

Twelve ionomer membranes were cut from the same sheet of IonPower Nafion N117 (nominal thickness 177.8 *μ*m) with a length of 85 ± 0.5 mm. Six had a nominal width of 5 ± 1 mm, while the other six had a width of 40 ± 1 mm. First the membranes were cut, and they underwent a 30-minute bath sonication using a Branson 1510 ultrasonic cleaner to remove possible impurities. After cleaning, they were soaked in a 1 M NaCl (aqueous) solution for 24 hours to promote ion exchange. After these treatments, the membranes were each tagged on one end with an identification number, and on the other end, a small piece of duct tape was attached to facilitate deflection measurements with a laser displacement sensor. The mass of the reflective tape (0.006 ± 0.002 g and 0.016 ± 0.003 g for the thin and wide membranes, respectively) was negligible compared to the mass of the membrane (0.174 ± 0.013 g and 1.378 ± 0.051 g for the thin and wide membranes, respectively). The samples were stored in deionized (DI) water while they were not tested.

### Experimental setup

**Figure 7:**
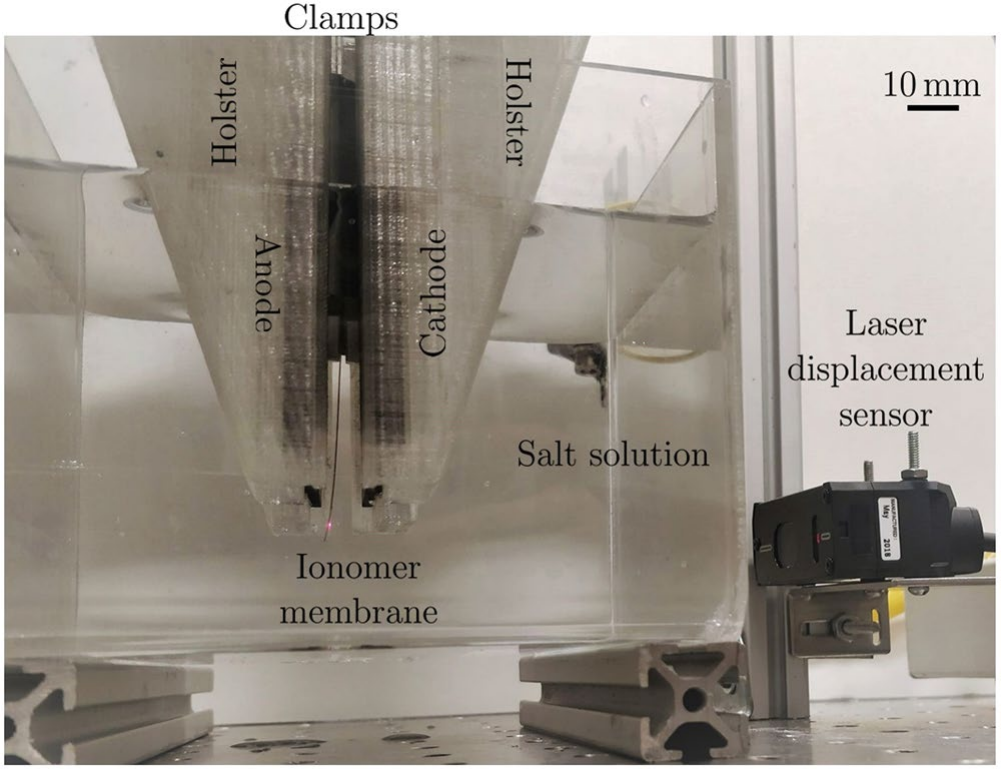
Picture of the experimental setup during a trial, showing a cantilever thin membrane between the two graphite electrodes, immersed in salt solution. The holsters and clamps suspending the electrodes and the membrane, respectively, are fixed to an aluminum frame, not shown in the picture. The laser displacement sensor points at the tip of the membrane, covered with tape to allow reflectance.

**Figure 8:**
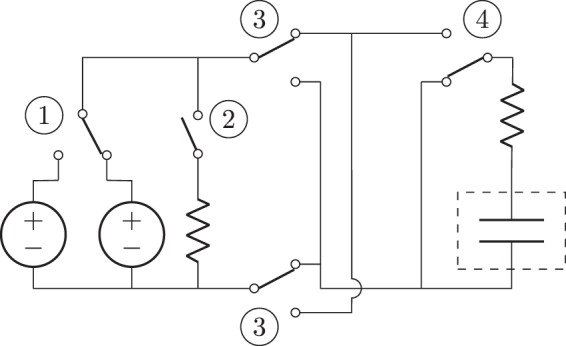
Schematics of the circuit used to generate a three-level square wave, with two levels of amplitude, where the plates in the dashed box represent the graphite electrodes. The switches are relays controlled by an Arduino Uno in a separate circuit.

During experiments, the ionomer samples were positioned between two parallel plate graphite electrodes (130 × 90 × 5 mm, 6 mm apart) in the center of a clear acrylic box (120 × 120 × 100 mm) filled with one liter of salt solution, as shown in Fig. 7. When the electrodes were submerged, the solution reached a height of 77.5 ± 0.5 mm. The graphite electrodes had a nominal wet surface area of 4000 ± 100 mm^2^ and were held by 3D printed holsters made from clear polylactic acid (PLA). The electrodes were connected via copper tape and alligator clamps to the driving circuit shown in Fig. 8, which generated the input signal for the experiments. To improve electrical contact with the graphite electrodes and reduce oxidation, the copper tape was placed between the electrodes and the 3D printed holsters above the solution. Two 3 mm thick acrylic plates were used to clamp the samples and kept them at a constant height during the experiments. Throughout the study, the free length of the samples was held constant at 45 mm, with 35 mm between the electrodes and 10 mm below. The clamp protruded underwater and raised the solution level, reducing the free wet surface area of the electrodes to 3100 ± 100 mm^2^. A laser displacement sensor (Keyence IL-100 with an IL-1500 signal amplifier) was secured to an aluminum beam and positioned perpendicular to the lateral surface of the samples. The laser was incident 2 mm above the bottom tip of each sample and output data at a rate of 3,000 Hz. We reduced the full-scale range of the laser to 1/20^th^ of its default value to improve the signal-to-noise ratio of the output signal.

The control circuit, shown in Fig. 8, used single pole double throw relays (SPDT) in conjunction with a double pole double throw relay (DPDT) to create a three-level square wave with a varying pulse amplitude. An Arduino Uno controlled each 5 V relay through an independent circuit. Switch 1 was used to determine the magnitude of the applied voltage by switching between two BK Precision 9110 100 W Multi-Range DC power supplies with a common ground. One of the power supplies was set to 1 V, and the other to 0.5 V, so that we could select between two input levels. To reduce the latency of the power supply and better simulate a step input voltage, switch 2 preloaded the selected power supply on a 10 Ω resistor, before it was connected to the electrodes. During a trial, switch 2 was shorted for ten seconds, then opened 10 ms before switch 4 connected the electrodes to the power supplies. Switch 3 was a DPDT, utilized to control the polarity of the signal. Each time the electrodes were disconnected from the power supplies, switch 3 reversed the polarity of the input signal. Last, switch 4 either shorted the electrodes or connected them to the power supply. A step pulse was delivered for one minute, separated by equal intervals of one minute when the electrodes were shorted and the polarity of the signal was inverted. From the circuit, we measured the voltage accross the electrodes and voltage across a 1 Ω resistor in series with the electrodes to obtain the current through the solution. All the measurements, including readings from the laser, were acquired by a NI 6221 data acquisition device (DAQ) at a uniform rate of 5,000 Hz per channel using LabView 2017.

### Laser calibration

Since the refractive index of salt water depends on the concentration^66^, we performed experiments to calibrate our laser displacement sensor in 0.1 M NaCl and 0.5 M NaCl. The laser was first set to a scaling factor of 1 and clamped against the clear acrylic box filled with solution (length 120 mm). We cut nine rectangular pieces of white 3 mm thick acrylic and created distance markers by stacking them. The thickness of each stack was measured with a digital caliper (resolution 0.01 mm) and was considered as the ground truth. The stacks were then positioned against the wall opposing the laser, and the voltage output of the laser for a given distance was found from the mean value of the voltage over ten seconds of measurement. Since the voltage output and distance are linearly related, we performed a linear fit between the thickness and mean voltage measurements. In the 0.1 M solution, the slope of this linear fit, which represents the conversion factor between voltage and distance, was 5.33 ± 0.03 mm/V (adjusted *R*^2^ = 0.9998), while in the 0.5 M solution, the slope was 5.15 ± 0.03 mm/V (adjusted *R*^2^ = 0.9997). Since we adjusted the scaling factor while measuring the ionomer tip displacement, this number was divided by 20 to account for the amplification of the signal.

### Experimental procedure

We considered all conditions listed in Table 1. For each solution concentration, we tested two thin and two wide membranes, washed the electrodes and the holsters with DI water to remove salt deposits from previous tests, replaced the copper tape to limit oxidation, and finally changed the solution to hinder contamination and evaporation. Overall, we tested the membranes in six solutions, three per each solution condition. A 0.1 M or 0.5 M NaCl solution was prepared by first sampling either 5.844 g or 29.22 g, respectively, of NaCl salt (Sigma-Aldrich S7653). DI water was mixed with the salt in a 2,000 mL beaker to obtain 1,000 mL of solution, which was then stirred at 700 rpm for five minutes. Finally, the solution was poured into the acrylic box, and the graphite electrodes were positioned in the center of the box.

For each trial, the sequence of 1 V and 0.5 V pulses from the circuit was determined a priori by a random number generator in Matlab 2015b. 20 pulses were considered in total, ensuring that the number of 1 V and 0.5 V pulses was equal for each polarity. The first trial was without any membrane, and was used as a control condition, performed with the clamp between the electrodes. After collecting data for the solution, a membrane was placed in the acrylic insert with a free length of 45 ± 0.5 mm. The acrylic insert was taped to ensure proper clamping, then placed between the electrodes. Once the membrane was placed, a new random number set for the circuit was generated and the trial started, with the DAQ collecting measurements. This procedure was repeated for each of the four samples tested with that solution. To conclude each set of experiments, one final set of measurements was collected without any membrane to ensure that the solution did not change over time, such that the mean of the peak current at 0.5 V did not vary more than one standard deviation.

### Data preprocessing

Using the time traces of the one-minute intervals of the pulses generated during each trial, a script in Matlab 2018a was used to calculate the peak current, the total charge stored at the electrodes, the peak displacement, and the time to reach the peak displacement for each condition. In some trials, the tip displacement of the sample was affected by a low-frequency drift in addition to high-frequency noise.

First, the beginning and end of each pulse were identified using the peaks of the current as delimiters. Due to the short time scale of the charge dynamics, the time at which the voltage was applied was used as the time at which the peak current was reached, and therefore the latter was taken as the origin of each interval. The peak current was found as the maximum current in the first second after the application of voltage. Then, the point at which the current decays to 5% of its peak value was determined using smoothed data, calculated with a moving average over 40 points to reduce the effect of noise. The region between the peak current and point of 5% decay was integrated using the trapezoidal method to obtain the total charge stored at the electrodes.

Since we separately considered the one-minute intervals of each pulse during a trial, the tip displacement time trace was offset by subtracting the average value of all data points one second before the peak current. To mitigate high frequency noise, we applied a Butterworth infinite impulse response filter (IIR)^67^ to the time trace of the tip displacement on a time lapse of six seconds (one second before the peak current and five seconds after). The IIR filter was set with a passband between 1–2 Hz and a cut-off band between 2–6 Hz, and its parameters were manually tuned to ensure the expected behavior in each interval. The value of the peak displacement and the time at which it was reached were determined from the filtered time traces.

### Statistical model

The datasets with the explanatory (width, concentration, and voltage) and response variables (peak current, total charge stored at the electrodes, time to reach the peak displacement, and peak displacement) were analyzed in R^68^. Each dataset was associated with the membrane identification number, to avoid pseudoreplication of the results^53^. The explanatory variables were considered as categorical variables. We fitted the peak current, total charge stored at the electrodes, and time to reach the peak displacement with a generalized linear mixed-effects model with Gamma error^53^, while we fitted the peak displacement with a linear mixed-effects model^53^, using the functions “glmer” and “lmer” from the “lme4_1.1-21” package^69^, respectively.

The use of a GLMM was necessary to normalize the residuals, due to the skewness of data distributions for the first three response variables (see Figs 3–5). In the model, the membrane number was used as a random effect. For each response variable, we started by fitting a model with the explanatory variables of each hypothesis and with full interactions, and we applied backward elimination^53^ to simplify it, selecting the most parsimonious model by minimizing the AICc^54^. For example, for peak displacement, the full model encompassed the applied voltage (required to test hypothesis H5), its pairwise interactions with the width and concentration, and the triadic interaction between all the three explanatory variables, while reduced models included the model with the applied voltage only and models with all possible combinations of pairwise interactions encompassing the applied voltage. In other words, we computed the AICc (with the function “AICc” from the “MuMin_1.43.6” package^70^) for each model obtained by the full model removing interactions, and we selected the one with the lowest value of the AICc as the most parsimonious.

From this procedure, we obtained the most parsimonious model that provides a complete description of the response variables with the least number of explanatory interactions (Table 2). For the peak current, hypotheses H1, H2, and H3 suggested a full model with all three explanatory variables and interactions between them. According to AICc, the most parsimonious model comprised the three variables with the interaction between solution concentration and applied voltage. For the total charge, the full and the most parsimonious models mirrored the ones of the peak current. The time to reach the peak displacement required the least complex model, whereby, from a full model with width and all the interactions comprising width for hypothesis H4, width and its interactions with concentration and voltage were the only explanatory variables in the most parsimonious model. For the peak displacement, we started from a full model with the voltage and all the interactions comprising voltage for hypothesis H5, and we retained the full model as the most parsimonious through AICc.

Once the most parsimonious model was obtained, we performed the ANOVA test^53^ using the “Anova” function from the “car_3.0-2” package^71^ that employs type II Wald Chi-squared tests to find the *p*-values for each explanatory variable and interaction present in the most parsimonious model (Table 2). Post-hoc analyses were performed through Tukey’s HSD^53^ by means of the function “emmeans” in the “emmeans_1.3.4” package^72^ to identify significant differences between conditions and elucidate whether the response variable decreased or increased with the explanatory variable. While performing post-hoc comparisons, the datasets were aggregated over those explanatory variables that did not present any significant effect and interaction from ANOVA tests. Throughout the statistical analysis, the significance level was set to 0.05.

## Supplementary information

Supplementary material

## Data Availability

Datasets and codes used in the analyses are stored at the authors’ home institution and will be provided on request.
