# Supplementary material for: Contactless actuation of perfluorinated ionomer membranes in salt solution: an experimental investigation
Source: Sci Rep. 2019 Aug 19;9:11989. doi: 10.1038/s41598-019-48235-9 (PMC6700092; doi:10.1038/s41598-019-48235-9)
Supplement: Supplementary file 1 — Supplementary material [file 41598_2019_48235_MOESM1_ESM.pdf]

Supplementary Information - Contactless actuation of perfluorinated ionomer membranes in salt solution: an experimental investigation

Alain Boldini[1], Maxwell Rosen[1], Youngsu Cha[2], and Maurizio Porfiri[1,3,*]

[1]Department of Mechanical and Aerospace Engineering, Tandon School of Engineering, New York University, 6 MetroTech Center, Brooklyn, NY 11201, USA
[2]Center for Intelligent & Interactive Robotics, Korea Institute of Science and Technology, Seoul 02792, Republic of Korea
[3]Department of Biomedical Engineering, Tandon School of Engineering, New York University, 6 MetroTech Center, Brooklyn, NY 11201, USA
[*]mporfiri@nyu.edu

Control experiments with deionized water and mylar membranes

In addition to the experimental and control conditions presented in the main text, we performed supplementary control tests on ionomer membranes in deionized (DI) water and mylar membranes in salt solution. These trials were used to confirm the necessity of the salt solution to actuate ionomer membranes and the impossibility to actuate ion-blocking membranes in the electrochemical cell.

In total, we tested two ionomer membranes in a solution of DI water, and two mylar membranes in 0.1 M and 0.5 M NaCl solutions. For the tests in DI water, we used thin and wide Nafion membranes from the main experiments. The trials with mylar were performed with two samples of nominal thickness 0.04 mm and dimensions 85×5 mm and 85×40 mm, identical to the "thin" and "wide" ionomer membranes, respectively.

Trials in DI water were performed using the experimental setup and procedure described in the main text, simply substituting the salt solution with DI water. Experiments on mylar membranes also followed the same experimental procedure of the main text, whereby we performed, for each solution, a first trial without any membrane, a trial with the thin mylar membrane, a trial with the wide mylar membrane, two trials with thin and wide ionomer membranes (to ensure consistency with respect to data in the main text), and a final trial without any membrane.

These supplementary conditions are listed in Table S1 and S2. In these tables, we present the mean value with standard error of the peak current, total charge stored at the electrodes, peak displacement of the sample, and time to reach the peak displacement, defined in the main text.

In DI water, we always registered a negligible peak current through the electrodes. Similarly, the total charge stored at the electrodes was negligible. Consistent with our intuition that current is related to the peak displacement, the measured peak displacement was less than $1/100^{\text{th}}$ of recordings in the main text on ionomer membranes. We attribute the observed motion to measurement noise.

The tests with mylar membranes were more interesting, especially with respect to the electrochemical variables. The peak current showed a peculiar dependence on the presence and the width of the membrane, which interacted with the concentration. For the same solution concentration and applied voltage, peak current and charge of trials with the thin membrane or without any membrane were within one standard deviation. However, for wide membranes, we noted a decrease in the peak current for 0.1 M, while no change was registered for the higher concentration – these changes were not mirrored by the stored charge that seemed unaffected. This could suggest that the possibility of ion motion along multiaxial branches is possible only for sufficiently large concentrations. Similar to ionomer membranes in DI water, mylar membranes did not show any appreciable motion, suggesting that the ion selectivity of the ionomer is important for actuation.

**Conditions**		**Features extracted**			
Width	Voltage [V]	Peak current [A]	Charge [C]	Time to reach peak displacement [s]	Peak displacement [μm]
Solution	0.5	0.0000 (0.0001)	0.0000 (0.0000)	NA	NA
Solution	1	0.0003 (0.0001)	0.0019 (0.0019)	NA	NA
Thin	0.5	0.0003 (0.0004)	0.0023 (0.0023)	2.2004 (0.2844)	0.0971 (0.025)
Thin	1	0.0004 (0.0002)	0 (0.0000)	1.9363 (0.2299)	0.171 (0.0283)
Wide	0.5	0.0004 (0.0003)	0.0000 (0)	1.8484 (0.2775)	0.0492 (0.0258)
Wide	1	0.0001 (0.0003)	0.0000 (0.0000)	2.018 (0.2044)	0.1701 (0.0596)

Table S1. Mean values and standard errors (in parentheses) of the response variables for each experimental condition with DI water. NA indicates not available data, corresponding to the four control conditions in the absence of the ionomer membrane. Note that standard errors are computed over twenty trials for the solutions and over ten trials for the membranes.

Conditions			Features extracted			
Width	Molarity [M]	Voltage [V]	Peak current [A]	Charge [C]	Time to reach peak displacement [s]	Peak displacement [μm]
Solution	0.1	0.5	0.0614 (0.0003)	0.0055 (0.0001)	NA	NA
Solution	0.1	1	0.1405 (0.0006)	0.0138 (0.0002)	NA	NA
Solution	0.5	0.5	0.0851 (0.0004)	0.0069 (0.0001)	NA	NA
Solution	0.5	1	0.2073 (0.0005)	0.0163 (0.0002)	NA	NA
Thin	0.1	0.5	0.0608 (0.0003)	0.0055 (0.0001)	0.4262 (0.092)	3.0682 (0.7018)
Thin	0.1	1	0.1401 (0.0003)	0.0132 (0.0002)	0.3917 (0.0925)	1.8674 (1.5932)
Thin	0.5	0.5	0.0856 (0.0005)	0.0068 (0.0001)	0.3382 (0.066)	3.6502 (0.9801)
Thin	0.5	1	0.2055 (0.0009)	0.0163 (0.0002)	0.3417 (0.0726)	2.8608 (1.0419)
Wide	0.1	0.5	0.0565 (0.0014)	0.0056 (0.0001)	0.4344 (0.0811)	1.6018 (0.8114)
Wide	0.1	1	0.1282 (0.0039)	0.0136 (0.0002)	0.3339 (0.0752)	2.8647 (1.3805)
Wide	0.5	0.5	0.0824 (0.0011)	0.0068 (0.0001)	0.3669 (0.0645)	2.6036 (1.298)
Wide	0.5	1	0.1997 (0.002)	0.0162 (0.0002)	0.3408 (0.0611)	2.0405 (1.1074)

Table S2. Mean values and standard errors (in parentheses) of the response variables for each experimental condition with mylar membranes. NA indicates not available data, corresponding to the four control conditions in the absence of the mylar membrane. Note that standard errors are computed over twenty trials for the solutions and over ten trials for the membranes.
